# Adaptation of evidence-based approaches to promote HIV testing and treatment engagement among high-risk Nigerian youth

**DOI:** 10.1371/journal.pone.0258190

**Published:** 2021-10-06

**Authors:** Lisa M. Kuhns, Amy K. Johnson, Adedotun Adetunji, Kehinde M. Kuti, Robert Garofalo, Olayinka Omigbodun, Olutosin A. Awolude, Bibilola D. Oladeji, Baiba Berzins, Ogochukwu Okonkwor, Oluwaseun P. Amoo, Omolade Olomola, Babafemi Taiwo

**Affiliations:** 1 Ann & Robert H. Lurie Children’s Hospital of Chicago, Chicago, IL, United States of America; 2 Department of Pediatrics, Feinberg School of Medicine, Northwestern University, Chicago, IL, United States of America; 3 Department of Family Medicine, University College Hospital, Ibadan, Nigeria; 4 HIV Program, Infectious Disease Institute, College of Medicine, University of Ibadan, Ibadan, Nigeria; 5 Centre for Child and Adolescent Mental Health, College of Medicine, University of Ibadan, Ibadan, Nigeria; 6 Department of Psychiatry, College of Medicine, University of Ibadan, Ibadan, Nigeria; 7 Department of Child and Adolescent Psychiatry, University College Hospital, Ibadan, Nigeria; 8 Department of Obstetrics and Gynecology, College of Medicine, University of Ibadan, Ibadan, Nigeria; 9 Division of Infectious Diseases and Center for Global Health, Northwestern University, Chicago, IL, United States of America; 10 Department of Private and Property Law, Faculty of Law, University of Ibadan, Ibadan, Nigeria; South African Medical Research Council, SOUTH AFRICA

## Abstract

**Background:**

Nigeria has the second highest number of people living with HIV (PLWH) globally, and evidence-based approaches are needed to achieve national goals to identify, treat, and reduce new infections. Youth between the ages of 15–24, including young men who have sex with men (YMSM), are disproportionately impacted by the Nigerian HIV epidemic. The purpose of this study was to inform adaptation of evidence-based peer navigation and mHealth approaches (social media outreach to promote HIV testing; short messaging service text message reminders to promote HIV treatment engagement) to the local context within iCARE Nigeria, a multi-phase study designed to investigate combination interventions to promote HIV testing and care engagement among youth in Nigeria.

**Methods:**

To elicit expert and community perspectives, a local group of advisors from academia, community, and governmental sectors provided feedback on intervention adaptation, which then informed a series of focus groups with stakeholders in Ibadan, Nigeria. Focus group data were collected over a period of three days in December of 2018. Participants in focus groups included YMSM and HIV-positive youth in care ages 16–24, and HIV service providers from local AIDS service organizations (ASO). Groups were stratified by HIV serostatus, gender, and stakeholder type. Focus group sessions were conducted using a semi-structured interview guide, audio-recorded, transcribed verbatim, and analyzed using a content analysis approach.

**Results:**

Local experts recommended intervention adaptations specific to the status of peer navigators as volunteers, peer characteristics (slightly older age, high maturity level, HIV/YMSM status), and intervention characteristics and resources (low navigator to peer ratio; flexible matching by demographic and social characteristics; social media platforms and content). Five focus group discussions with stakeholders, including 27 participants were conducted to elicit feedback on these and other potential adaptations. Youth participants (n = 21) were mean age 20 years (range = 16–24); 76% HIV-positive, 76% men and 48% MSM. Service providers (n = 6) represented both HIV prevention and care services. Participants across stratified subgroups reported largely positive perceptions and high perceived acceptability of both mHealth and peer navigation strategies, and echoed the recommendations of the advisory group for volunteer-based navigators to promote altruism, with a low navigator-peer ratio (1:5). Participants emphasized the need to incorporate minimal mobile data use strategies and popular social media platforms among YMSM (e.g., Facebook, Grindr) for widespread access and reach of the interventions.

**Conclusions:**

In Ibadan, Nigeria, stakeholders support the adaptation of combined mHealth and peer navigation strategies to promote HIV testing and care engagement among high-risk youth. Recommended adaptations for the local context reflect concerns about the feasibility and sustainability of the intervention and are expected to improve accessibility and acceptability.

## Introduction

Nigeria has more than 1.9 million people living with HIV/AIDS [1], the second highest in the world; however, it has been largely neglected as a focus of public health efforts compared to other “hot spots” in sub-Saharan Africa (SSA) [2]. In 2018, Nigeria had 130,000 new HIV infections and 53,000 AIDS-related deaths [1]. Progress towards UNAIDS 95-95-95 goals in Nigeria has been slow, with an estimated 67% of people living with HIV aware of their status, 55% on antiretroviral therapy (ART), and 42% virally suppressed [3].

The United Nations defines youth as individuals aged 15–24 in recognition of the developmental risks to health and well-being of mid-to-late adolescence and young adulthood [4]. Nigerian youth ages 15–24 are disproportionately impacted by HIV. At the front end of the HIV care continuum, rates of HIV testing among youth in Nigeria are low (<25% ever tested), and more so for young men in comparison to young women [5]. A World Health Organization (WHO) study suggests that fear (e.g., of family/community reaction or life disruption) among youth is a major barrier to HIV testing, with levels of fear twice as high in young men who have sex with men (YMSM) [6]. Among youth at high risk for HIV infection, YMSM are a group of particular concern due to widespread discrimination and stigmatization likely driving the epidemic in this group. MSM are the only group in which HIV prevalence is still rising in Nigeria with an estimated 23% prevalence, the highest rate of all risk groups [7]. YMSM are hard-to-reach and engage for prevention in Nigeria due in part to the Same-Sex Marriage Prohibition Act of 2014 [8, 9], which bans same-sex “amorous relationships” with penalties of up to 14 years in prison. YMSM also experience barriers similar to other youth in Nigeria, including fear of being diagnosed HIV-positive and related stigmatization, and lack of access to youth-friendly testing services [10]. In a critical review of current global efforts to promote HIV testing among youth, including YMSM and other key populations, providing testing outside of traditional healthcare facilities (community-based, youth center, club, drop-in testing), and making these services youth-friendly, are key recommendations [11].

There are also critical challenges to promote treatment engagement and viral suppression among youth living with HIV (YLH) in Nigeria. According to UNAIDS [12], Nigeria is one of the six countries with half of the global burden of youth (15–19 years) living with HIV. An estimated 190,000 youth ages 15–24 in Nigeria are living with HIV, with 28,000 new infections in 2019. In 2019, an estimated 3,100 youth, ages 15–24 died of AIDS-related causes in Nigeria [7]. Transition to adult HIV care routinely begins at age 15, however few HIV care centers have adolescent-specific clinics and comprehensive transition services are rare [13]. Factors associated with poor HIV-related outcomes among youth in Nigeria, and across SSA, include stigma and lack of disclosure to others, low social support, and less availability of youth-specific services [14–17]. In particular, medication adherence, a key driver of disease progression [18], is lower among youth in comparison to adults in SSA, with a lack of evidence-based interventions to address it [14].

Peer support interventions are recommended by the WHO as a strategy that provides many benefits for youth across the HIV care continuum [6] and shifts supportive care to community-based and non-professional providers for efficient use of resources [19, 20]. Peer-based HIV supportive interventions are based on both social cognitive and social support theories [21]. Peers can be an important source of developmentally-grounded psychological and social support, helping to build confidence, resilience, reducing anxiety and promoting a sense of belonging [6, 19, 21]. Peers share personal characteristics, circumstances and/or experiences [19], which can help YLH to cope with fear, hopelessness, stigma and discrimination, and facilitate problem-solving. Peer support can also motivate and reinforce self-care and help to build self-efficacy for HIV testing and treatment [6]. For YMSM, heightened stigma and rejection by family members and the larger community may undermine care engagement, making peer support particularly helpful [22]. Peer support includes a focus on enhancing motivation, building skills and problem solving techniques delivered either in-person or via mobile devices by peers who have been trained in HIV-related topics, leadership skills, setting boundaries and maintaining confidentiality [23].

Technology-based strategies have emerged as a promising approach to HIV-related interventions, particularly among youth, with a growing body of evidence suggesting efficacy [24–26]. mHealth is a generic term that applies broadly to electronic interventions delivered on mobile devices, including via short messaging service (SMS) text and web-based applications (apps) [27]. Although not fully utilized, mHealth approaches may be feasible in Nigeria given high teledensity (over 100%), according to the Nigerian Communication Commission [28], and increasing rates of both smartphone and social media use, particularly among youth [29]. Approaches using mHealth have had positive effects across the HIV care continuum and have the advantage of simple interfaces for users, accessibility anywhere cell signals and/or Wi-Fi are available, relative affordability, and have been promoted specifically to reach stigmatized and disenfranchised populations [30, 31]. Interventions using mHealth approaches in SSA have primarily focused on support for antiretroviral therapy (ART) adherence with evidence of feasibility and efficacy [32–36], but have not been widely evaluated among youth across the HIV care continuum. One SMS text message reminder intervention (designed to address “forgetting”), the Treatment Text intervention (TXTXT), has shown evidence of both feasibility and efficacy to promote ART adherence among YLH in the US [37], but has not yet been tested in SSA. TXTXT is a bi-directional and personalized reminder intervention based on social cognitive theory [38, 39] to promote medication adherence, including incorporation of environmental cues to action and self-management support and feedback.

Social media outreach is an evidence-based mHealth strategy to promote HIV testing, although it has not been evaluated among YMSM in SSA. It is based on theories of empowerment education, social cognitive theory and natural helping by peers [40]. Implementation occurs in a format that mirrors common HIV prevention practice targeting days of the week and hours in which YMSM are most likely to be communicating and using mobile devices. Peer health educators create public social media profiles and post educational information of interest to the focal population to generate followers and facilitate referral to HIV testing [40]. In a review of social media approaches to promote HIV testing globally (primarily among MSM), outreach via social media platforms was efficacious in increasing HIV testing; effects on HIV testing increased for interactive (two-way messaging) versus passive (one-way, static messaging) approaches [25] and for those with peer-based interactions [41, 42]. Using peer-based, participatory approaches may enhance the effectiveness and relevance of HIV-related social media activities, increasing motivation and support for completion of prevention activities, such as testing for HIV. Use of existing social media platforms, in particular, is a practical and efficacious approach to increase HIV testing [40, 42–44].

In sum, while there is an urgent need to curb the HIV/AIDS epidemic among Nigeria’s youth, particularly YMSM, evidence-based approaches that hold promise have not been systematically evaluated in the country. The Intensive Combination Approach to Rollback the Epidemic in Nigerian Adolescents (iCARE Nigeria) is an initiative to adapt and test two combination interventions, each of which include peer navigation and mHealth components, to promote HIV testing and HIV treatment, respectively, among youth in Nigeria. The HIV testing intervention, focused on YMSM, combines social media outreach with peer navigation to promote testing and linkage to HIV care, while the HIV treatment intervention, focuses on all YLH, and combines an SMS medication reminder approach, TXTXT, with peer navigation to promote HIV treatment engagement. We conducted formative research to adapt these intervention approaches to the Nigerian environment prior to planned feasibility and efficacy studies.

## Methods

### Overview

The iCARE Nigeria study is a collaborative partnership between investigators at Northwestern University and Ann & Robert H. Lurie Children’s Hospital (Lurie Children’s) in Chicago, Illinois, in the United States (U.S.) and from the College of Medicine (COM), University of Ibadan (UI), and University College Hospital (UCH), Ibadan, Nigeria. Our approach to adaptation of the peer navigation and mHealth approaches was drawn from best practices for adaption of public health interventions [45], as well as those specific to HIV prevention and treatment [46–48] including distilling components deemed to be adaptable (i.e., not core behavior change techniques), and seeking feedback on adaptation from both local experts and stakeholders. The consolidated criteria for reporting qualitative research (COREQ) were followed for the reporting of focus group findings detailed herein (see COREQ Checklist, S1 Table) [49].

#### Expert consultation

Our investigative team distilled key components of each intervention approach from peer-reviewed literature and implementation guides a priori, with a particular focus on distinguishing between core intervention characteristics or behavior change techniques, and other intervention elements that would be potentially adaptable to cultural, social, or logistical conditions [50]. We then consulted with local experts in academic, legal, community and governmental sectors to inform intervention adaptation. Feedback from advisors was provided in an all-day face-to-face meeting, recorded in detailed notes. These notes were reflected back to the group in real time to build consensus and confirm recommendations to inform the structure and content of the focus group interview guide, which was used to obtain feedback from local stakeholders regarding adaptation of the intervention approaches.

#### Stakeholder focus groups

Key stakeholders included YMSM (including HIV-negative/status unknown and HIV-positive young men in separate groups), YLH (including both young men and young women in separate groups), and representatives of AIDS Service Organizations (ASOs). The YMSM and YLH represented potential intervention participants. Focus groups with stakeholders were stratified based on HIV serostatus, gender, and/or stakeholder type. Participants were known to the study investigators in Ibadan as clinic patients or youth seeking HIV preventative services and were selected and approached purposively by them, based on representativeness of the population. Focus groups were held at the Institute of Infectious Disease Institute, College of Medicine, University of Ibadan (IDI/CoMUI) over a 3-day period, December 18–20. Ibadan is the third-largest city in Nigeria and thus reflects a mid-size urban environment. Group discussions were facilitated in English by at least one investigator from Nigeria and one from the US in each group, using a semi-structured interview guide. The YMSM groups were facilitated by a team of one male and one female-identified facilitator (AA, LMK), the YLH groups by two female facilitators (KMK, AJK) and the ASO group by two male facilitators (AA, BT), who are all faculty members at their respective academic institutions with training in qualitative data collection. A notetaker was also present in each group to record contemporaneous notes. Notetakers were drawn from bachelor’s or master’s prepared iCARE study staff of the IDI/CoMUI, who received training for note taking and qualitative data collection in preparation for these tasks. The interview guide included questions related to experiences with HIV testing, linkage to care, and treatment (among YLH and YMSM) to contextualize the interventions, followed by specific questions about the proposed intervention components (per recommendations by the expert advisors), and the role of staff training, including what types of training were most important (see Interview Guides, S1 File). Focus group sessions were audio-recorded, and transcribed verbatim. Due to the one-time nature of the focus group sessions and anonymity of reported data, transcripts and findings were not shared with participants for correction or comment. Transcripts were uploaded into Dedoose, a cloud-based mixed-methods software, for analysis [51]. Data were then analyzed using a rapid content analysis approach to inform the elaboration of intervention design, procedures manuals and training materials [52]. A preliminary codebook was structured based on the interview guide and by reviewing a transcript selected at random; all coders (N = 2) reviewed and revised the preliminary codebook. Next, the codebook was applied by the primary coder to two transcripts. The secondary coder coded a subset of excerpts selected at random by Dedoose and achieved reliability at >.80 measured by Cohen’s kappa [53]. Most divergences occurred due to omission of a sub-code and upon review were quickly rectified to 100% agreement. After achieving reliability, coders met to discuss modifying codes resulting in condensing multiple codes and adding three new codes. All transcripts were then coded using the updated codebook and were reviewed by both coders for consensus of code application (see Coding Tree, S1 Fig). Finally, themes were created based on clustering of code application.

The Institutional Review Boards of Northwestern University, Lurie Children’s and UI/UCH Ethical Review Committee provided ethical approval for study procedures. Focus group participants provided written informed consent; the consent forms described the purpose of the study and procedures.

## Results

### Expert consultation

The expert advisory group met with members of our investigative team in an all-day meeting in mid-December of 2018 in Ibadan Nigeria. The group included 8 experts from governmental entities (e.g., State AIDS Program, Department of Public Health); community-based organizations focused on human rights issues (including sexual minority concerns) and youth reproductive health; a legal consultant, and collaborating medical providers and investigators at partner universities in the largest city in Nigeria, Lagos, which is in the south and Jos, a mid-size city, which is in the north. The legal expert on the panel advised on intervention design in the context of the Same-Sex Marriage Prohibition Act, i.e., to avoid incriminating staff or participants. Advisory group members were drawn from local community-based organizations serving youth and YMSM, in particular, and academic institutions in Ibadan, as well as from national youth sexuality and health and a national youth advocacy organization. Collaborators from partner universities were medical providers with expertise in HIV prevention and care.

The basic core elements of each intervention approach, drawn from published literature and implementation guides a priori by the investigative team, are listed in Table 1. These core components, defined as those that are key to produce desired outcomes [54], were described to the advisory group as part of their orientation to the feedback session. For peer navigation (for both HIV testing and HIV treatment support), these include being peer-led (peer defined as sharing common characteristics) [21, 25], delivered in patient or participant-centered format [6, 19], and empowerment-focused in terms of behavior support and change [6, 22]. For the ART text message reminder intervention, core characteristics include personalized messages, bidirectional messaging, messages timed with daily dosing, and directed to youth themselves for behavior change [37, 55]. For the social media outreach, core elements included using a flexible approach (dynamic and loosely structured), responsive to social media subscriber input [40], using a sexual health-education based approach (not solely focused on HIV or HIV testing) [40], and interactive posting/messaging (not passive) [25] to encourage and motivate HIV testing.

**Table 1:** Core intervention elements, iCARE Nigeria.

Intervention Type	Core Elements
Peer navigation	• Peer-led • Participant-centered • Empowerment-focused
mHealth: Text message medication reminders	• Personalized • Bi-directional • Timed with daily dosing • Directed to youth
mHealth: Social Media Outreach	• Flexible and responsive • Sexual health approach • Interactive (not passive)

We sought feedback from local experts on the aspects of each intervention that were potentially adaptable to the local culture (see Table 2). Adaptable intervention elements are those, for example, that reflect use of language, implementation methods, and contextual considerations [56]. For the peer navigation interventions, this included employment classification (i.e., employee vs volunteer); peer characteristics (e.g., eligibility and peer-to-peer matching criteria); as well as intervention characteristics (e.g., ratio of navigator-to-peer). In terms of employment classification, advisors recommended that peer navigators in each intervention be classified as volunteers, rather than regular staff and be paid a small stipend to cover navigation-related costs, rather than being salaried. In terms of peer characteristics, they recommended that, for the HIV treatment intervention, peer navigators should be HIV-positive and virally suppressed or clinically stable according to their primary care provider. They suggested matching navigators to peers based on flexible criteria that could include gender, religion, and employment or student status. In addition, for the HIV testing intervention, they recommended that peers should be primarily YMSM themselves, but also include non-YMSM for comfort in navigation to testing or treatment among more fearful or “closeted” individuals. For both interventions, they suggested that navigators be a few years older than the target age groups to serve as experienced role models. In terms of intervention characteristics, given the suggestion that peers be volunteers, they recommended a relative low ratio of navigator to peers (1: <10).

**Table 2:** Core and adaptable intervention elements with expert and stakeholder recommendations, iCARE Nigeria.

Intervention Type	Adaptable Elements	Recommended Adaptations—Experts	Recommended Adaptations- Stakeholders in Focus Groups
**Peer navigation** *	• Employment status and compensation	• Volunteer-based • Stipend (not salary)	• Volunteer-based • Stipend to offset costs
	• Peer navigator characteristics (eligibility, navigator-to-peer matching criteria)	• Slightly older than peers • Role models • HIV-positive and virally suppressed or doing well (HIV treatment intervention) • YMSM or allies (HIV testing intervention) • Match navigator-to-peer based on gender, religion, employment, student status	• Slightly older than peers • Role models • Trustworthy, tolerant • HIV-positive or knowledgeable about HIV (for both interventions) • Primarily YMSM (HIV testing intervention)
	• Intervention characteristics (ratio of navigator-to-peer)	• Low ratio of navigator to peers (1:<10)	• Low ratio of navigator to peers (1:5) • Trained in the basics of HIV infection and treatment • Trained in the use of resources • Provide with condoms (HIV testing intervention)
**mHealth: Text message medication reminders** (HIV treatment)	• Content of encouraging messages (“canned”)	• Navigators review encouraging messages	• Consider affordability of devices and data • Protect confidentiality
**mHealth: Social Media Outreach** (HIV testing)	• Choice of social media platform • Health education topics	• Instagram, Twitter, WhatsApp, Grindr • Safety and human rights training • Use low data consumption approach	• Facebook, WhatsApp, Grindr • Protect confidentiality and promote safety

*Each of the two interventions includes a peer-navigation component, in addition to the mHealth component.

For the mHealth strategies, intervention characteristics were most pertinent. For the TXTXT medication reminder intervention, the primary intervention characteristic for adaptation was considered to be the content of the “canned” encouraging messages. Advisors suggested a review of messages by the peer navigators to update and adapt them for saliency to local youth.

For social media outreach, intervention characteristics for adaptation included the choice of social media platform(s) as well as the choice of health education topics for social media messaging. Advisors recommended the use of Instagram, Twitter, WhatsApp and Grindr as their impression of the most popular among YMSM. Also, they recommended incorporating safety measures and training in human rights for the peer navigators in the HIV testing intervention given the criminalization of homosexuality in Nigeria. Advisors further recommended reviewing platforms carefully for consumption of data and using those that were most data efficient.

### Focus groups

Following the feedback sessions with experts, five focus groups (one each: HIV-positive young men, HIV-positive young women, HIV-positive YMSM, HIV-negative/status unknown YMSM, and ASO representatives) were then held with a total of 27 stakeholders. All youth who expressed interest in participation consented for participation and completed the entire focus group session. Youth participants (n = 21) were mean age 20 years (range = 16–24); 76% HIV-positive (n = 16), 76% men (n = 16) and 48% MSM (n = 10; 5 HIV-positive, 5 HIV negative/unknown). Service providers (n = 6) represented both HIV prevention and care services. One focus group consisting of young women living with HIV (n = 5) was not captured via audio recording due to equipment failure, and therefore only the contemporaneous notes were used for subsequent analysis. Focus group sessions lasted approximately 2 hours.

Participants were asked their opinions on intervention strategies, with a focus on acceptability, key characteristics, recommendations, and perceived challenges. Participant comments are grouped below as related to peer navigation and mHealth intervention components and quotes are identified by group type (YMSM, YLH, ASO). Content analysis revealed that, overall, the intervention strategies were perceived as universally acceptable, feedback was largely positive, and recommendations largely supported those provided by the expert advisory group. Participants noted the intervention components (peer navigation; text messaging; social media outreach) were useful (5 of 5 focus groups), familiar (5), desirable (4), and were perceived as impactful (4) and needed (5).

#### Peer navigation

Specifically, for the peer navigation interventions, participants understood and were familiar with the concept of a peer navigator, which they also called a “supporter” or a “peer supporter.” Focus group participants unanimously agreed that peer navigation was a useful strategy that appealed to them, indicating high acceptability. Participants highlighted trust, confidence, honesty, as well as common experiences and attitudes as important characteristics in peer navigators. They also discussed desired characteristics of assigned peers, including personality (tolerance, respect for others), age, HIV-status, and sexual orientation:

*Because we know what we are feeling… we have the same feelings, so we know how we are…. We can share more ideas than what we are going through… We know that since we are both, we are all in the same shoes. So, that is a good idea. (YLH young men’s group)*

*We start with qualities having trust and confidence in people, if I get to a community and I am not practicing what I am preaching… the person must be a role model, trust always there for me, people in the community need people that can trust so you don’t push them, very accommodating, another thing is influence the decision of peers really matters a lot (ASO group)*

For MSM-identified participants, they felt peer navigators should also be from the MSM community:

*If the person is in the community, there’s going to be a very big, like a large, big bond that is going to be established between the peers and the Peer Navigator.*

*(YMSM, HIV-negative/unknown group)*

In terms of other characteristics, participants had mixed opinions on whether or not the peer navigator should be the same or opposite sex, living with HIV, or if the HIV status was not important as long as the peer navigator was well informed about HIV. Participants discussed the importance of the peer navigator’s age and agreed that peers should be somewhat older and more mature with salient lived experience, yet cautioned against peers that are parental or “too old.” Discussions centered around finding the balance between a peer who is mature, yet does not seem old:

*I think inasmuch as I want to listen to somebody that is my age that understands what’s going on currently, I would also want to listen to somebody that has seen a lot of other things that I have not seen. So, I think it should be a combination, but I think it should be tilted towards the young side; but still within the large, wide age range. (YMSM, HIV-negative/unknown group)*

*I don’t know…people don’t tend to go for the older people—it would be ridiculous to make your peer your daddy (YMSM, HIV-negative/unknown group)*

In terms of intervention characteristics, although many ranges were discussed, ultimately the ratio of peer-to-navigator was thought to be best at 4 to 5 peers per navigator. This was uniformly agreed to in all focus groups:

*I can’t chat for more than four people at a time…I actually thought about it like how. Let me just say five. (YMSM, HIV-positive group)*

*For now five. For now, maybe later run sha four [“Maybe four later” in colloquial Nigerian English]. (YMSM, HIV-positive group)*

In terms of employment status, participants across groups generally agreed that volunteer navigators would be better than employees, with a caveat regarding coverage of basic costs they may incur. For example, one person said:

*Ok. I think that, like he said, passion is the main thing that should drive anyone that wants to be part of the project, because if you are enticing the person with money, you will not get the best out of the person. But if it is…if it’s passion-driven, then you are assured of the best from whoever that is going to volunteer. (YMSM HIV-negative/unknown group)*

Another participant outlined the caveat for this:

*If you look at basically, it’s…there are a lot of costs involved in maybe messaging, calling and all of that. So, I think, inasmuch as we want to say it is voluntary and all of that, a lot of people still have issues with finances. So, I think, if -maybe not paying, like paying as per wage-…I think helping them with cost, like to deal with the cost of the job is also another thing. Then, asides that, payment doesn’t necessarily have to be in cash; there could be other things you could do—certifications, certain privileges they have as a result of the work they are doing and all that kind of things. I think it’s very important when people see things from beyond money perspective (YMSM HIV-negative/unknown group)*

Participants were asked about necessary training components for peer navigators. All groups discussed the need to train peer navigators on basic HIV prevention, treatment, and health promotion information:

*I think that one of the things the Peer Navigator should learn is effects of ARV, because if they eventually get positive clients, they…you know, depending on the person, they might experience so many things. (YMSM, HIV-negative/unknown group)*

*The Peer Navigator should equip himself with information, because when you have information, you can… prepare for everything; (YLH male group)*

Participants in one focus group highlighted the role of religious beliefs that may impact the work of a peer navigator. Participants stressed the importance of understanding these beliefs and providing information in support of continuing medication and treatment for HIV. To illustrate, this participant describes how people may test positive but believe that they can be cured via church or their religious beliefs:

*Because there are a whole lot of stories I’ve heard about people we are in Nigeria; we are very religious in this nation that when somebody tests positive and they just tell you when next you see them maybe you’re like following them up they’ll tell you “I’ve gone to the church and my pastor tells me that I’ free. So, I don’t have HIV in my bloodstream again.” And some people will tell you that “it’s the blood of Jesus that is running in my vein.” So, the blood of Jesus does not contain virus and the likes. (YMSM, HIV-positive group)*

Participants discussed beyond training, that peer navigators should be knowledgeable about resources and understand how to refer peers to mental health support, medical services, food services, have access to condoms, lubricant, and HIV test kits (for the HIV testing intervention). One participant stated, and others in the group agreed, about the importance of peer navigators distributing condoms:

*give them [peer navigators] condoms, lubricants, able to encourage safe sex habits; like that’s also very important…like, give them condoms. Because one of the reasons why people don’t use condoms is because there is no condom, or they are shy to buy it outside. (ASO group)*

#### SMS text intervention

With regard to the mHealth interventions, when asked about daily personalized text message reminders to promote medication adherence, participants responded positively and unanimously supported the idea:

*I actually support the SMS, because I myself, I am very frequent with SMS a lot. If you go with calling. People will feel like who is this person calling. That keeps calling you everyday. And for SMS…luckily my phone, there is a way I can hide a particular SMS from a person. You won’t be able to see it. I go with the SMS. Once the SMS enters, the phone will just ring. But calling…people will feel who is this person calling. If he calls you in the morning and call you at night…they will say who is this person? They might even get provoked one day and would just collect the phone from me. At least I want to know that person. But with the SMS that one nobody will know the person. Just check your phone. (YLH young men’s group)*

Despite supporting the text messaging intervention, participants had concerns about barriers including lack of airtime on mobile phones, cracked screens on mobile phones that limit ability to read SMS, loss or theft of phones, and concerns around privacy/confidentiality of the SMS.

*I support the SMS because it will be on your phone and you need to check. And again, for subscription issue, not everyone can afford that. (ASO group)*

*I foresee a challenge especially young people some of these young people are minors still supervised by the parents, ask who sent you this message, how safe are these young ones, compose messages that does not expose them had to change some of my messages at a point (YMSM, HIV-negative/unknown group)*

Two focus groups mentioned engaging parents and caregivers with text messages to remind youth living with HIV to take medication. In the young women’s group, one participant was concerned that a parent or caregiver might forget to deliver the message or even check the messages at all. In the second group, all participants felt like engaging parents was a good idea, yet some had preferences for receiving SMS themselves:

*Will that SMS reminders be to you or may be to a parent that’s going to release you to come?*

*Although they have our contacts, they still have our parents. They still have to tell our parents but it is advisable to send it to them…what I’m saying is that it is advisable to send it to the students, some students will just ignore. But, if you send it to parents, parents will remind you that you are to come, but as for me, I prefer the student. (YLH young men’s group)*

Focus group participants were also asked about pre-programmed SMS encouraging responses. Participants like the idea of using emoticons or the phrases “thumbs up,” “good going,” and “good job”. Participants also suggested using coded language that would match the reminder SMS:

*make it more fun with our own jargons have you kissed your pillow tonight, have you said your prayer tonight ……especially for young persons …………the other part to it when they get that you have to get a feedback from them and an encouraging response what kind of response? Ride on, if he says no, prayer is the key. If he says yes, ride on. (ASO group)*

#### Social media outreach

Finally, participants were asked to provide feedback on the social media outreach strategy with a particular focus on identifying common websites used and any potential perceived challenges. Prior to starting the discussion about social media outreach, facilitators asked a general question about what could be done to increase HIV testing. In one focus group participants immediately suggested utilizing social media due to widespread acceptability:

*Facilitator: But to increase the number of young people testing now, what do you think we need to do?*

*Participant: Ah…probably…well, maybe the use of these applications we use─ give them more information…*

*Facilitator: What applications?*

*Participant: Grindr, Facebook, or any other source*

*Facilitator: For HIV testing?*

*Participant: Yes, to make them aware─*

*(YMSM, HIV-positive group)*

Participants reported that Facebook is a very common platform and suggested its use in outreach:

*Facebook messenger is like number one…So I think apart from Grinder, Facebook is also another important place. But Facebook doesn’t give you any pop-ups like that too. (YMSM, HIV-positive group)*

Participants indicated the ubiquitous nature of Grindr use among YMSM, in particular:

*Facilitator: let me say it this way: out of…if we say out of…if we put down on the average, if we put down ten people who are YMSM, how many people would be comfortable with Grindr…who will be familiar with Grindr, out of ten?*

*Participant1: at least 8*

*Participant2: 8 people, and then the remaining will definitely not have a phone*

*[everyone bursts into laughter]*

*Facilitator: So practically everybody who has a phone knows of Grindr. (YMSM, HIV-positive group)*

Aside from endorsing the use of social media platforms and listing the most commonly used sites, participants also stressed privacy and safety in using social media for outreach. These concerns stemmed largely from YMSM who wanted to ensure their privacy would be protected. In this exchange, participants discuss a blogger who breached the privacy of a Facebook group and posted private messages from YMSM online, exposing their identity:

*Participant 1: really that place…I think…to me, I think it’s not secure, because, you know, Facebook doesn’t have the security─ for MSM, I’m saying…one can successfully…*

*Participant 2(interrupts): one group on Facebook─ they had issues lately when somebody updated his sexual experience and then a blogger took the message and then posted it online. So, since when…So I think the guy…*

*Facilitator: with his identity?*

*Participant 2: Yes. So since then they took the account of the Admin of the group and then the group was sort of secured to an extent—you can’t be in the group unless somebody adds you…unless like a member of the group adds you… (YMSM. HIV-positive group)*

Participants shared that they preferred phones that had biometric security features such as thumbprint locks. However, this was also countered saying that friends or family will just ask that you unlock your phone so they can use it. Participants stated they used apps that concealed their messages and pictures received in order to maintain privacy of their social media use.

## Discussion

In this study, we sought feedback to adapt evidence-based peer navigation and mHealth approaches to promote HIV testing and HIV treatment engagement, respectively, among youth at-risk of and living with HIV in Nigeria. Local experts helped to identify points of adaptation, while community stakeholders provided feedback on both the feasibility and acceptability of the intervention approaches, as well as the adapted elements.

Our local experts recommended adaptations to peer navigation for both the HIV testing and treatment interventions that largely reflect concerns about feasibility and sustainability. Recommendations to use volunteers who receive a stipend, instead of employees who earn a salary, reflects a concern about resources to sustain the interventions over time. Furthermore, while prior HIV treatment interventions, in particular, have adopted a 1:10 ratio of navigator-peers [57], this was perceived as unsustainable for a volunteer force. These findings are consistent with concerns among stakeholders across SSA [58], and in Nigeria more specifically [59], about efficiencies in systems of HIV care given reduced funding from external donors, which have impacted the quantity [60] and quality of HIV-related service provision [61], and care engagement [62]. An umbrella review of 39 systematic reviews of the effectiveness of volunteer workers concluded that they were as good as or better in supporting positive outcomes for a variety of health conditions, however, they were not as good at complex tasks, such as diagnosis and counselling. The review suggests that while promising, the use of volunteers for community health promotion would be strengthened by careful training and implementation, strong policy backing and continual support from managers [60]. Thus, while using a volunteer workforce may be an adaptation to peer navigation that saves costs, training and on-going support is of high importance for successful implementation.

Recommendations regarding peer navigator characteristics and matching of navigator to peers reflect the potential salience of identity and related experiences (e.g., YLH, YMSM), as well as the importance of gender and religion in the local culture for reaching and successfully intervening with the focal groups of youth. Among YMSM specifically, there were concerns about the risk to peer navigators given the criminalization of homosexuality, thus the recommendation for human rights training to promote their safety.

Comments from stakeholders in focus groups validate key aspects of peer navigation and mHealth intervention approaches. Much like the local experts, they agreed that common experiences and understanding among peers may help to promote both HIV testing as well as treatment engagement, which is consistent with recommendations for HIV-related intervention with youth [6] and YMSM in particular [22]. In addition, their comments largely echoed the recommendations of the expert advisory group for designating a volunteer status for navigators, with recommendation of a ratio of 4–5 peers to each navigator. They also agreed that navigators should be slightly older than intervention participants and be knowledgeable and seen as role models. This endorsement of older role model status for navigators, across both the expert advisors and stakeholders, reflects in part, the underlying hierarchy of Nigerian society, respecting both age and position. Among stakeholders, recommendation for volunteer status was tied to the importance of altruistic motivation (rather than sustainability), which was perceived as reflecting true motivation to help others. The recognition of the key role of peers, as described by focus group participants, reflects common facilitators of sustainability in SSA more broadly, including “community ownership,” “working within existing resources,” and “community buy-in through volunteerism,” as articulated in a recent review of facilitators and barriers to sustainability of public health interventions in SSA [63].

Regarding the mHealth components of each intervention, stakeholders largely endorsed the ubiquity of both text messaging and social media use among the focal groups of youth, i.e., HIV-positive and YMSM respectively, with some feasibility caveats. Concerns about these approaches included confidentiality on the one hand, and potential limitations to technology access among some youth due to cost of both devices and data, on the other. Both text messaging and social media messaging present opportunities for breaches of confidentiality of HIV and/or YMSM status. Thus, they recommended approaches to promote access, as well as to protect confidentiality [64].

Additional comments by focus group participants highlight recommended delivery mechanisms, message content, and platforms. While some participants suggested messages might be delivered to parents or caregivers in addition to, or instead of, the youth themselves, this suggestion was not widespread and may undermine a core characteristic of the TXTXT intervention: to support youth empowerment and self-management. Participants largely endorsed another core characteristic of the TXTXT intervention, personalization of messages, indicating that these may increase both salience of the messages and confidentiality. Finally, focus group participants endorsed the primary social media platforms suggested by local advisors, including WhatsApp and Grindr to reach YMSM, as well as Facebook.

A key recommendation of stakeholders was appropriate training of peer navigators, particularly in confidentiality and professionalism, but also in the medical management of HIV infection to counter commons myths. This was perceived as ensuring promotion of positive relationships between navigator and peers, as well as confidence in recommended actions.

Limitations for this study include the single-session feedback session with our advisory group, which may have limited the breadth and depth of expert advice, the relatively small sample size for focus group feedback and technical problems with the focus group recording among HIV-positive young women, which may have limited coverage of salient themes related to each intervention among the sample as a whole, and young women in particular. In reviewing detailed notes recorded for the young women’s group, we did not find additional salient themes, although notes are not as comprehensive as verbatim transcripts. In addition, conducting the focus groups in English may have limited the contributions of some youth who speak English as a second language. While the limited sample size did not allow us to reach saturation in subgroups, consistency in findings across these groups suggests cross-group saturation of themes. In addition, the high level of correspondence between recommendations of local experts and stakeholders reinforced confidence for saturation of themes.

## Conclusions

We conclude that in Ibadan, Nigeria, local experts and stakeholders support adaptation of mHealth and peer navigation strategies to promote HIV testing and care engagement among high-risk youth. Recommended adaptations reflect concerns about feasibility and sustainability, as well as effectiveness and safety in the local context, which are necessary to maximize accessibility and acceptability. The adapted interventions are being investigated at multiple clinical research sites across Nigeria.

## Supporting information

S1 Table COREQ 32-ITEM checklist. (DOCX)

S1 File Focus group guides. (DOCX)

S1 Fig Focus group data coding tree. (TIF)
