# Supplementary material for: Adaptation of evidence-based approaches to promote HIV testing and treatment engagement among high-risk Nigerian youth
Source: PLoS One. 2021 Oct 6;16(10):e0258190. doi: 10.1371/journal.pone.0258190 (PMC8494297; doi:10.1371/journal.pone.0258190)
Supplement: S1 Table — (DOCX) [file pone.0258190.s001.docx]

**S1 Table**

**S1. COREQ 32-ITEM Checklist**

Tong A, Sainsbury P, Craig J (2007 Consolidated criteria for reporting qualitative research (COREQ): a 32-item checklist for interviews and focus groups. International Journal for Quality in Healthcare: 19-349-357

| No. Item | Guide questions/description | Reported on Page # |
| --- | --- | --- |
| 1. Interviewer/facilitator | Which authors conducted the focus groups? | p.9 |
| 2. Credentials | What were the researchers’ credentials? | p.9 |
| 3. Occupation | What was their occupation at the time of the study? | p.9 |
| 4. Gender | Was the researcher male or female? | p.9 |
| 5. Experience and training | What experience or training did the researcher have? | p.9 |
| 6. Relationship with participants established | Was a relationship established prior to study commencement? | p.8 |
| 7. Participant knowledge of the interviewer | What did the participants know about the researcher? | p.10 |
| 8. Interviewer characteristics | What characteristics were reported about the interviewer/facilitator? | p.9 |
| 9. Methodological orientation and theory | What methodological orientation was stated to underpin the study? | p.5-7 |
| 10. Sampling | How were participants selected? | p.8 |
| 11. Method of approach | How were participants approached? | p.8 |
| 12. Sample size | How many participants were in the study? | p.14 |
| 13. Non-participation | How many people refused to participate or dropped out? Reasons? | p.14 |
| 14. Setting of data collection | Where were the data collected? | p.9 |
| 15. Presence of non-participants | Was anyone else present besides the participants and researchers? | p.9 |
| 16. Description of sample | What are the important characteristics of the sample? | p.14 |
| 17. Interview guide | Were questions, prompts, guides a provided by the authors? | p.9 and Appendix A |
| 18. Repeat interviews | Were repeat interviews carried out? | N/A |
| 19. Audio/visual recording | Did the research use audio or visual recording to collected the data? | p.9 |
| 20. Field notes | Were field notes made during and/or after the focus groups? | p.9 |
| 21. Duration | What was the duration of the focus groups? | p.14 |
| 22. Data saturation | Was data saturation discussed? | p.25 |
| 23. Transcripts returned | Were transcripts returned to participants for comment and/or correction? | p.9 |
| 24. Number of data coders | How many data coders coded the data? | p.9 |
| 25. Description of the coding tree | Did authors provide a description of the coding tree? | p. 11 and S3 Appendix Coding Tree |
| 26. Derivation of themes | Were themes identified in advance or derived from the data? | p.9 |
| 27. Software | What software, if applicable was used to manage the data? | p.9 |
| 28. Participant checking | Did participants provide feedback on the findings? | p.9 |
| 29. Quotations presented | Were participant quotations presented to illustrate the themes/findings? Was each quotation identified? | p.14 |
| 30. Data and findings consistent | Was there consistency between the data presented and the findings? | p.21 |
| 31. Clarity of major themes | Were major themes clearly presented in the findings? | p.15-21 |
| 32. Clarity of minor themes | Is there a description of diverse cases or discussion of minor themes? | p. 15-21 |
