# Supplementary material for: Adaptation of evidence-based approaches to promote HIV testing and treatment engagement among high-risk Nigerian youth
Source: PLoS One. 2021 Oct 6;16(10):e0258190. doi: 10.1371/journal.pone.0258190 (PMC8494297; doi:10.1371/journal.pone.0258190)
Supplement: S1 File — (DOCX) [file pone.0258190.s002.docx]

**S2 File**

**S2. Focus Group Guides**

**iCare Nigeria**

**Focus Group Interview Guide – Care Engagement**

*[Instructions to interviewer: Participants must complete the consent process prior to initiating the interview.]*

Facilitator: Good Morning (or afternoon) everyone! You are welcome to this discussion group. My name is ……. (FILL IN). You are already very familiar with me because I am involved with your care but I would like to introduce some colleagues from the University College Hospital, Ibadan, and Lurie Children’s Hospital, Northwestern University, Chicago [FILL IN]. We all work together in a team.

We are interested in hearing from you and learning from you. We want to hear about your experiences with HIV care services. We also want to obtain your views and opinions about how to improve HIV care services among youth, aged 15-24 years. We want to use the information we obtain from you to develop new interventions to promote HIV testing and engagement in care among young people.

Now that you know a little bit about us, we can start. The first thing we are going to have you do is write your name on that card in front of you. Write whatever you would like us to call you during the discussion, you can use a nickname or a name that is not even your real name for this meeting.

When you are done with that, please open the envelope in front of you, and complete the questions about yourself. Please let us know if you need some help with completing these questions. Please ask us questions about this and if you want one of us to write the things down for you, let us know. We will provide a private place for this. We would like you to complete every single question. This is very important. When you have finished answering every single question to the best of your ability, please place it back in the envelope and pass it to me. Thank you.

We would like to explain some things about our discussion today before we get started:

- - The audio recording is for research purposes and will be used by only the researchers. When we are done, someone will type out every word that was said. After that, we will destroy the recordings so that everyone’s participation is unknown (anonymous).
  - We will be asking lots of questions. There are no right or wrong answers. We will ask you to explain your answers in detail. For example, we may ask a lot of “Why?” questions. This is so we can fully understand your thoughts. Please feel free to express yourselves. Again I stress that no one will judge your responses and there are no right or wrong answers.
  - Everything we say in this room is considered confidential. Please do not share what you hear today with anyone else. We want you to feel comfortable telling us your thoughts and opinions.
  - Be respectful of each other’s answers and allow each other a chance to speak.
  - If you are being very quiet, we may ask you specifically what you think of a certain topic, if at any time you do not feel like speaking, just say so.
  - If you are sharing a lot, we may ask you to hold your thoughts for a moment to encourage others to share as well. We want to hear from everyone, and some of us need more encouragement than others.
  - Please turn off your mobile phones now.
  - We encourage you to try and sit through the entire period of discussion. If you need to use the toilet, call or text someone, please step out quietly and re-join as soon as possible.
  - Do you have questions or comments?

*[Instructions to facilitator: answer any questions the participants may have.]*

**HIV care experiences**

**General**

Facilitator**:** We are looking into ways to improve HIV care for young people like you. HIV care may mean many things to different people so we want to be sure we are all thinking about the same things. By HIV care we mean all the care and support that young people receive from an HIV service to stay healthy and happy despite living with HIV, which includes support to register in and stay connected to an HIV treatment center, obtain medications for HIV, taking the medications, keeping clinic appointments and having blood taken regularly to monitor treatment and health.

We would like to start off the group by hearing your experiences with HIV care.

Can you describe your experiences with HIV care as a young person?

What are the difficulties you face with receiving HIV care as a young person?

What are the typical challenges to receiving HIV care as a young person?

*Probes:*

*Discrimination, Disclosure, Access, Transportation; Finances, Personal issues, Family issues*

*Religious concerns, Community, Health services*

What aspects of the HIV care do you have concerns about as a young person?

Why do you have concerns about this?

(if no concerns) Can you tell us what aspects you think might need improvement?

What are some things that might make it easier for a young person, such as you, to receive HIV care?

Can you tell me about the aspects of HIV care that you are happy about?

**Engagement and Retention in HIV Care**

What factors affect your willingness to seek help (engagement) for HIV care as a young person?

*Probes:*

*Discrimination, Disclosure, Access, Transportation; Finances, Personal issues, Family issues*

*Religious concerns, Community, Health services*

What factors affect your willingness to continue to receive and remain in HIV care (retention) as a young person?

*Discrimination, Disclosure, Access, Transportation; Finances, Personal issues, Family issues*

*Religious concerns, Community, Health services*

Have you heard that if your viral load is undetectable that you are very unlikely to transmit HIV to other people when you have sex with them?

*Does this influence your behaviour? Does this make you more likely to take your medication?*

**Questions about HIV Care**

Facilitator: Thank you very much for sharing!

Now we would like to get your advice and comments about a specific type of intervention that may enhance your willingness to seek help (promote engagement) in HIV care. This intervention would have the following aspects

- Other young people living with HIV (peers) would provide peer support, which means they will be involved in reaching out to you to encourage you to access care (promote engagement) and stay in HIV care (retention) once you have accessed care .
- A regular text message will be sent to you to encourage and support you to use your HIV medication regularly (HIV medication adherence) and keep your clinic appointments.

1. What are your views about using peers to support the willingness of young people living with HIV (PLHIV) to access HIV care (HIV care engagement)
2. What are the good things (advantages, pros) about this idea (peer support to help HIV care engagement)?
3. What are the not so good things (disadvantages, cons) about this idea (peer support to help HIV care engagement)?
4. Are there any other things you feel we should know about using peers to support access to care (HIV care engagement) for young people?

*Interviewer note: ensure balance of responses for pros/cons, ask each, elaborate, list back to ensure all captured for recording*

1. Do you think that using peer support is a good idea?
   1. What kind of personal or professional characteristics should we look for in hiring a peer supporter?

*Probe: age, sex, expertise, reputation, etc.*

- 1. What do you think the key tasks should be for the peer?

*Probe: frequency of contact, etc.*

- 1. If you were part of this program, receiving peer support, what would you like the peer to do?
  2. What are the potential challenges they would face to provide peer support?
     1. How can we address these challenges?
  3. Would you join a program like this as a peer supporter that is someone who is providing support to HIV-infected young people? What impacts your decision? Is there anything that could be done to change your decision? *(e.g., no: family does not want you to; yes: incentives )*

Part of this intervention is sending daily text messages to participants as a way to remind them to take their HIV medication every day and as a way to help keep HIV medical appointments.

1. What are your views about sending daily text messages to participants as a way to remind them to take their HIV medication every day?
2. Do you have any concerns about this strategy (sending daily text messages)?
3. What type of young person might this program work best for?
4. What are some challenges we might face in sending text messages to youth to remind them to take their medication and as a way to help keep HIV medical appointments?

*Probe: privacy? Phone service? Access to medication?*

- 1. If this program was offered to you, would you sign up? Why or why not? Is there anything that could change your decision?

1. For this intervention, we ask participants to text us back- when they respond that they have taken their medicine we send back a text message with a positive response- like “great job!” and “cool!”. What are examples of messages you think they would like to hear to encourage them to take their medication? Anything specific that a young person might relate to that old people like us don’t know about (e.g., words or phrases)?
2. Do you have other comments or questions about these interventions? Is there anything else we should know?

**iCare Nigeria**

**Focus Group Interview Guide – CBOs**

*[Instructions to interviewer: Participants must complete the consent process prior to initiating the interview.]*

Facilitator: Good Morning (or afternoon) everyone! You are welcome to this discussion. My name is……. (FILL IN) and I work at the University College Hospital. I would like to introduce my colleagues also from the University College Hospital, Ibadan and from the Lurie Children’s Hospital in Chicago and Northwestern University [FILL IN]. We are all working together as a team on this project.

As individuals who work in the community, we are interested in hearing your experiences and opinions about how to improve HIV testing and care services among youth, aged 15-24.

We are interested in hearing from you and learning from you. We want to hear about your experiences with HIV care services. We also want to obtain your views and opinions about how to improve HIV care services among youth, aged 15-24 years. We want to use the information we obtain from you to develop new interventions to promote HIV testing and engagement in care among young people.

Now that you know a little bit about us, we can start. The first thing we are going to have you do is write your name on that card in front of you. Write whatever you would like us to call you during the discussion, you can use a nickname or a name that is not even your real name for this meeting.

When you are done with that, please open the envelope in front of you, and complete the questions about yourself. Please let us know if you need some help with completing these questions. Please ask us questions about this and if you want one of us to write the things down for you, let us know. We will provide a private place for this. We would like you to complete every single question. This is very important. When you have finished answering every single question to the best of your ability, please place it back in the envelope and pass it to me. Thank you.

We would like to explain some things about our discussion today before we get started:

- - The audio recording is for research purposes and will be used by only the researchers. When we are done, someone will type out every word that was said. After that, we will destroy the recordings so that everyone’s participation is anonymous.
  - We will be asking lots of questions. There are no right or wrong answers. We will ask you to explain your answers in detail. For example, we may ask a lot of “Why?” questions. This is so we can fully understand you thoughts. Please feel free to express yourselves. Again I stress that no one will judge your responses and there are no right or wrong answers.
  - Everything we say in this room is considered confidential. Please do not share what you hear today with anyone else. We want you to feel comfortable telling us your thoughts and opinions.
  - Be respectful of each other’s answers and allow each other a chance to speak.
  - If you are being very quiet, we may ask you specifically what you think of a certain topic, if at any time you do not feel like speaking, just say so.
  - If you are sharing a lot, we may ask you to hold your thoughts for a moment to encourage others to share as well. We want to hear from everyone, and some of us need more encouragement than others.
  - Please turn off your mobile phones now.
  - We encourage you to try and sit through the entire period of discussion. If you need to use the toilet, call or text someone, please step out quietly and re-join as soon as possible.
  - Do you have questions or comments?

*[Instructions to facilitator: answer any questions the participants may have.]*

**HIV Care Experiences**

**General**

Facilitator**:** We are looking into ways to improve HIV care for young people. HIV care may mean many things to different people so we want to be sure we are all thinking about the same things. By HIV care we mean all the care and support that young people receive from a HIV service. This includes experiences with HIV counseling and testing, safer sex practices, prevention of HIV transmission, or staying healthy and happy despite living with HIV. It also includes obtaining medication for HIV, continuing to use medications as prescribed and having blood taken for testing.

We would like to start off the group by hearing your experiences with the provision of HIV counseling and testing, safer sex practices and prevention of HIV transmission, or staying healthy and happy despite living with HIV for young people in the community.

Can you describe your experiences with HIV care provision for young people in the community?

What are the difficulties young people face with receiving HIV care in the community?

What are the typical challenges young people have with receiving HIV care in the community?

*Probes:*

*Discrimination Disclosure Access Transportation Finances*

*Personal issues Family issues Religious concerns Community*

*Health services e.t.c.*

What aspects of the HIV care for young people do you have concerns about as a community worker?

Why do you have concerns about this?

(if no concerns) Can you tell us what aspects you think might need improvement?

What are some things that might make it easier for young persons with HIV to receive care in the community?

What aspects of HIV care for young people are you happy about?

(if yes) Can you tell me about these aspects?

**Engagement and Retention in HIV Care**

What factors would affect the willingness of young people to seek help (engagement) for HIV care?

*Probes:*

*Discrimination Disclosure Access Transportation Finances*

*Personal issues Family issues Religious concerns Community*

*Health services etc*

What factors would affect the willingness of young people to continue to receive and remain in HIV care (retention)?

*Probes:*

*Discrimination Disclosure Access Transportation Finances*

*Personal issues Family issues Religious concerns Community*

*Health services etc*

**HIV Testing**

The HIV testing approach we are planning to use is to increase access to testing among high-risk young men, in particular, so we will ask you questions specific to this group.

We would like to create an intervention or programme to increase rates of HIV testing among high-risk youth. What we know so far is that many youth avoid testing.

Why do you think young people in this environment avoid HIV testing?

*Probes:*

Could it be because they are afraid? Tell me what you know about this.

Could it be because they do not know where to go to get tested? Tell me all you know about this

Could it be that they do not want their parents or other family members or others to find out? Tell me what you know about this.

What are your views about these reasons? Do you agree with them?

What are some other reasons why a young person might not get tested?

*Probes:*

*Are there reasons or barriers to HIV testing that impact specific subgroups of young people*

*Young women?*

*Young men*

*Young Men who have sex with other men*

*Young sex workers*

*Young people who use drugs*

*Are there any other groups that are of concern? Tell me about them*

What type of testing services are available for young people?

*Probe:*

*By areas of the city*

What hours of the day are the HIV testing services (that you know of) available to users?”

What are the barriers or obstacles to accessing or using these services?

What do you think is needed to increase HIV testing among young people?

What are the best places in the community, to go for testing?

What makes some better than others?

How do young people find out about where to go for testing?

What types of testing are available?

*Probes:*

Community

Clinic-based

Rapid

Outreach services provided from health services and NGOs (provider initiated testing)

Services the young people seek out themselves (client initiated testing).

Where would one go for a confirmatory test (blood test to confirm HIV positive)?

Are there barriers to receiving a confirmatory test?

Have you heard about home or self-testing (get a test and take it home)?

Would you use home testing?

What are the potential barriers to home or self-testing?

*Probes:*

*Do you think patients would trust the results?*

*Would they be worried about other people seeing the test or results?*

*Do you think it would help promote regular testing if patients didn’t have to go somewhere to get tested?*

What would be the challenges to getting someone linked to HIV care if they tested at home?

**HIV Testing Intervention**

Facilitator: Thank you for that feedback. Now, we will like to get your advice about specific types of interventions to increase HIV testing. The interventions would include the following activities:

1. Text messages or the use of other aspects of the social media

2. Peer navigation to HIV testing and, if needed, to HIV care. By peer navigation, we mean having young people who are members of the same high-risk groups or who are HIV-infected, help other young people access HIV testing and care.

What forms of social media are you aware of?

What forms do young people commonly use in the environment?

What are the key social media platforms that we could use to reach young men in order to increase HIV testing?

*Probe:*

*What should we know about these social media platforms?*

*Is there already HIV or sexual health information connected to these platforms?*

What are the potential benefits of using social media to reach young men?

What are some of the drawbacks of using social media to reach young men?

What do you think of using peers for support and guidance to HIV testing (peer navigation) ?

Is it a good idea?

Is it a bad idea?

Does this type of programme already exist? (Probe: if so, describe key characteristics and successes)

- What kind of personal or professional characteristics should we look for in hiring a peer navigator?
- What do you think the navigator would need to do to help people get linked to testing or care (Probes: reminders, help with transportation, accompany to visits)?
- What are the potential challenges they would face to navigate individuals to testing services? What about to HIV care?
  - 1. How can we address these challenges?

Do you have other comments or questions about these interventions to increase HIV testing?

Is there anything else we should know?

*Probe: What kind of problems might we encounter? What might be a better idea?*

**Questions about HIV Care**

Facilitator: Thank you for that feedback. Now, I’d like to get your advice about a specific type of intervention to promote engagement in HIV care. The intervention would include peer support to promote HIV care, as well as text messages to support consistent daily use of HIV tablets as recommended (HIV medication adherence). This intervention will be available to all HIV-positive youth, regardless of whether or not they are male, female, transgender, or something else.

What are the potential positive and negative aspects of using peers to support HIV care engagement?

What kind of personal or professional characteristics should we look for in hiring a peer supporter? *Interviewer note: ensure balance of responses for pros/cons, ask each, elaborate, list back to ensure all captured for recording*

Do you think that using peers support is a good idea?

- What do you think the key tasks should be for the peer?
- What are the potential challenges they would face to provide peer support?
  - 1. How can we address these challenges?

Part of this intervention will involve sending daily text messages to participants as a way to remind them to take their HIV medication every day.

- What are some challenges we might face in sending text messages to youth to remind them to take their medication?
  - - *Probe: privacy? Phone service? Access to medication?*
- For this intervention, we ask participants to text us back- when they respond that they have taken their medication we send back a text message with a positive response- like “great job!” and “cool!”.
- What are examples of messages you think they would like to hear to encourage them to take their medication?
- Anything specific that a young person might relate to that people like us don’t know about (e.g., words or phrases?)?
- Do you have other comments or questions about these interventions?
- Is there anything else we should know?

**iCare Nigeria**

**Focus Group Interview Guide – Testing**

*[Instructions to interviewer: Participants must complete the consent process prior to initiating the interview.]*

Facilitator: Good Morning (or afternoon) everyone! You are welcome to this discussion group. My name is ……. (FILL IN). You are already familiar with me because I am involved with your care but I would like to introduce some other colleagues from the University College Hospital, Ibadan, and Lurie Children’s Hospital, Northwestern University, Chicago [FILL IN]. We all work together in a team.

We are interested in hearing from you and learning from you. We want to hear about your experiences with HIV testing and engagement in care (HIV care services). We also want to obtain your views and opinions about how to improve HIV care services among youth, aged 15-24 years. We want to use the information we obtain from you to develop new interventions to promote HIV testing and engagement in care among young people.

Now that you know a little bit about us, we can start. The first thing we are going to have you do is write your name on that card in front of you. Write whatever you would like us to call you during the discussion, you can use a nickname or a name that is not even your real name for this meeting.

When you are done with that, please open the envelope in front of you, and complete the questions about yourself. Please let us know if you need some help with completing these questions. Please ask us questions about this and if you want one of us to write the things down for you, let us know. We will provide a private place for this. We would like you to complete every single question. This is very important. When you have finished answering every single question to the best of your ability, please place it back in the envelope and pass it to me. Thank you.

We would like to explain some things about our discussion today before we get started:

- - The audio recording is for research purposes and will be used by only the researchers. When we are done, someone will type out every word that was said. After that, we will destroy the recordings so that everyone’s participation is unknown (anonymous).
  - We will be asking lots of questions. There are no right or wrong answers. We will ask you to explain your answers in detail. For example, we may ask a lot of “Why?” questions. This is so we can fully understand your thoughts. Please feel free to express yourselves. Again I stress that no one will judge your responses and there are no right or wrong answers.
  - Everything we say in this room is considered confidential. Please do not share what you hear today with anyone else. We want you to feel comfortable telling us your thoughts and opinions.
  - Be respectful of each other’s answers and allow each other a chance to speak.
  - If you are being very quiet, we may ask you specifically what you think of a certain topic, if at any time you do not feel like speaking, just say so.
  - If you are sharing a lot, we may ask you to hold your thoughts for a moment to encourage others to share as well. We want to hear from everyone, and some of us need more encouragement than others.
  - Please turn off your mobile phones now.
  - We encourage you to try and sit through the entire period of discussion. If you need to use the toilet, call or text someone, please step out quietly and re-join as soon as possible.
  - Do you have questions or comments?

*[Instructions to facilitator: answer any questions the participants may have.]*

**Questions about HIV Testing**

Facilitator: We will like to ask you some questions about your experiences with HIV testing, prevention and care services:

We would like to create an intervention or programme to increase rates of HIV testing among high risk youth.

What we know so far is that many youth avoid testing.

Why do you think young people avoid HIV testing?

*Probes:*

Could it be because they are afraid? Tell me what you know about this.

Could it be because they do not know where to go to get tested? Tell me all you know about this

Could it be that they do not want their parents or other family members or others to find out? Tell me what you know about this.

What are your views about these reasons? Do you agree with them?

What are some other reasons why a young person might not get tested?

*Probes:*

*Are there reasons or barriers to HIV testing that impact specific subgroups of young people*

*Young women?*

*Young men*

*Young Men who have sex with other men*

*Young sex workers*

*Young people who use drugs*

*Are there any other groups that are of concern? Tell me about them*

I would like to encourage us to discuss about our personal experiences with HIV Testing.

Have you ever been tested for HIV before? Tell us about this

Where did you go for testing?

*Probe: how did you choose this place over other options? How did you find out about it?*

What was good about it?

What was bad about it?

What would have made it a better experience?

*Probe: by areas of the city, open hours (after school, etc)*

What type of testing services are available for young people?

*Probe:*

*By areas of the city*

*When are these testing services open from?*

What type of testing services are available for young people?

What type of testing services are available for young men?

What type of testing services are available for young women?

What are the barriers or obstacles to accessing or using these services?

What do you think is needed to increase HIV testing among young people?

What are the best places in the community, to go for testing?

What makes some better than others?

How do young people find out about where to go for testing

What are the barriers or obstacles to accessing or using these services?

What do you think is needed to increase HIV testing among young people?

What do you think is needed to increase HIV testing among young men?

What do you think is needed to increase HIV testing among young women?

What types of testing are available?

*Probes:*

Community

Clinic-based

Rapid

Where would one go for a confirmatory test (blood test to confirm HIV positive)?

Are there barriers to receiving a confirmatory test?

Have you heard about home or self-testing (get a test and take it home)?

Would you use home testing?

What are the potential barriers to home or self-testing?

*Probes:*

*Do you think patients would trust the results?*

*Would they be worried about other people seeing the test or results?*

*Do you think it would help promote regular testing if patients didn’t have to go somewhere to get tested?*

What would be the challenges to getting someone linked to HIV care if they tested at home?

Have you heard that if you are diagnosed soon after you are HIV infected, and receive treatment soon after infection, that your chances of living longer and well with HIV are much better?

*How does this impact your behavior? Does it make you more likely to be tested and tested more often? Why or why not?*

**Questions about HIV Testing Intervention**

Facilitator: Thank you for that feedback. Now, I’d like to get your advice about a specific type of intervention to increase HIV testing. The intervention would include outreach via text messaging or social media, as well as peer navigation to HIV testing and, if needed, to HIV care. By peer navigation, we mean having young people who are members of the same high risk groups or who are HIV-infected, help other young people access HIV testing and care.

1. What are the key social media platforms that we could use to reach young men?

*Probe: what should we know about these social media platforms—is there already HIV or sexual health information connected to these platforms?*

1. What are the potential benefits of using social media to reach young men in order to increase HIV testing?
2. What are some of the drawbacks of using social media to reach young men?
3. Do you think that using peers for navigation (may need to define navigation) is a good idea?
   1. Does this type of program already exist?
   2. What kind of personal or professional characteristics should we look for in hiring a peer navigator?
   3. What do you think the navigator would need to do to help people get linked to testing or care (reminders, help with transportation, accompany to visits)?
   4. What are the potential challenges they would face to navigate individuals to testing services? What about to HIV care?
      1. How can we address these challenges?
4. Do you have other comments or questions about these interventions to increase HIV testing? Is there anything else we should know?

*Probe: would you take part in this type of intervention? What about your friends? What kind of problems might we encounter? What might be a better idea?*

1. Do you have other comments or questions about these interventions? Is there anything else we should know?

**HIV Testing Intervention**

Facilitator: Thank you for that feedback. Now, we will like to get your advice about specific types of interventions to increase HIV testing. The interventions would include the following activities:

1. Text messages or the use of other aspects of the social media

2. Peer navigation to HIV testing and, if needed, to HIV care. By peer navigation, we mean having young people who are members of the same high-risk groups or who are HIV-infected, help other young people access HIV testing and care.

What forms of social media are you aware of?

What forms do young people commonly use in the environment?

What are the key social media platforms that we could use to reach young men in order to increase HIV testing?

*Probe:*

*What should we know about these social media platforms?*

*Is there already HIV or sexual health information connected to these platforms?*

What are the potential benefits of using social media to reach young men?

What are some of the drawbacks of using social media to reach young men?

Do you think that using peers for navigation (may need to define navigation) to HIV testing is a good idea?

- Does this type of programme already exist? (probe: if so, describe key characteristics and successes)
- What kind of personal or professional characteristics should we look for in hiring a peer navigator?
- What do you think the navigator would need to do to help people get linked to testing or care (Probes: reminders, help with transportation, accompany to visits)?
- What are the potential challenges they would face to navigate individuals to testing services? What about to HIV care?
  - 1. How can we address these challenges?

Do you have other comments or questions about these interventions to increase HIV testing?

Is there anything else we should know?

*Probe: What kind of problems might we encounter? What might be a better idea?*

**Questions about HIV Care**

Facilitator: Thank you for that feedback. Now, I’d like to get your advice about a specific type of intervention to promote engagement in HIV care. The intervention would include peer support to promote HIV care, as well as text messages to support HIV medication adherence. This intervention will be available to all HIV-positive youth, regardless of whether or not they are male, female, transgender, or something else.

What are the potential pros and cons of using peers to support HIV care engagement?

*Interviewer note: ensure balance of responses for pros/cons, ask each, elaborate, list back to ensure all captured for recording*

Do you think that using peers support is a good idea?

- What kind of personal or professional characteristics should we look for in hiring a peer supporter?
- What do you think the key tasks should be for the peer?
- What are the potential challenges they would face to provide peer support?
  - 1. How can we address these challenges?

Part of this intervention will involve sending daily text messages to participants as a way to remind them to take their HIV medication every day.

- What are some challenges we might face in sending text messages to youth to remind them to take their medication?
  - - *Probe: privacy? Phone service? Access to medication?*
- For this intervention, we ask participants to text us back- when they respond that they have taken their medication we send back a text message with a positive response- like “great job!” and “cool!”.
- What are examples of messages you think they would like to hear to encourage them to take their medication?
- Anything specific that a young person might relate to that people like us don’t know about (e.g., words or phrases?)?
- Do you have other comments or questions about these interventions?

Is there anything else we should know?
