# Supplementary figures and images for: Adaptation of evidence-based approaches to promote HIV testing and treatment engagement among high-risk Nigerian youth
Source: PLoS One. 2021 Oct 6;16(10):e0258190. doi: 10.1371/journal.pone.0258190 (PMC8494297; doi:10.1371/journal.pone.0258190)

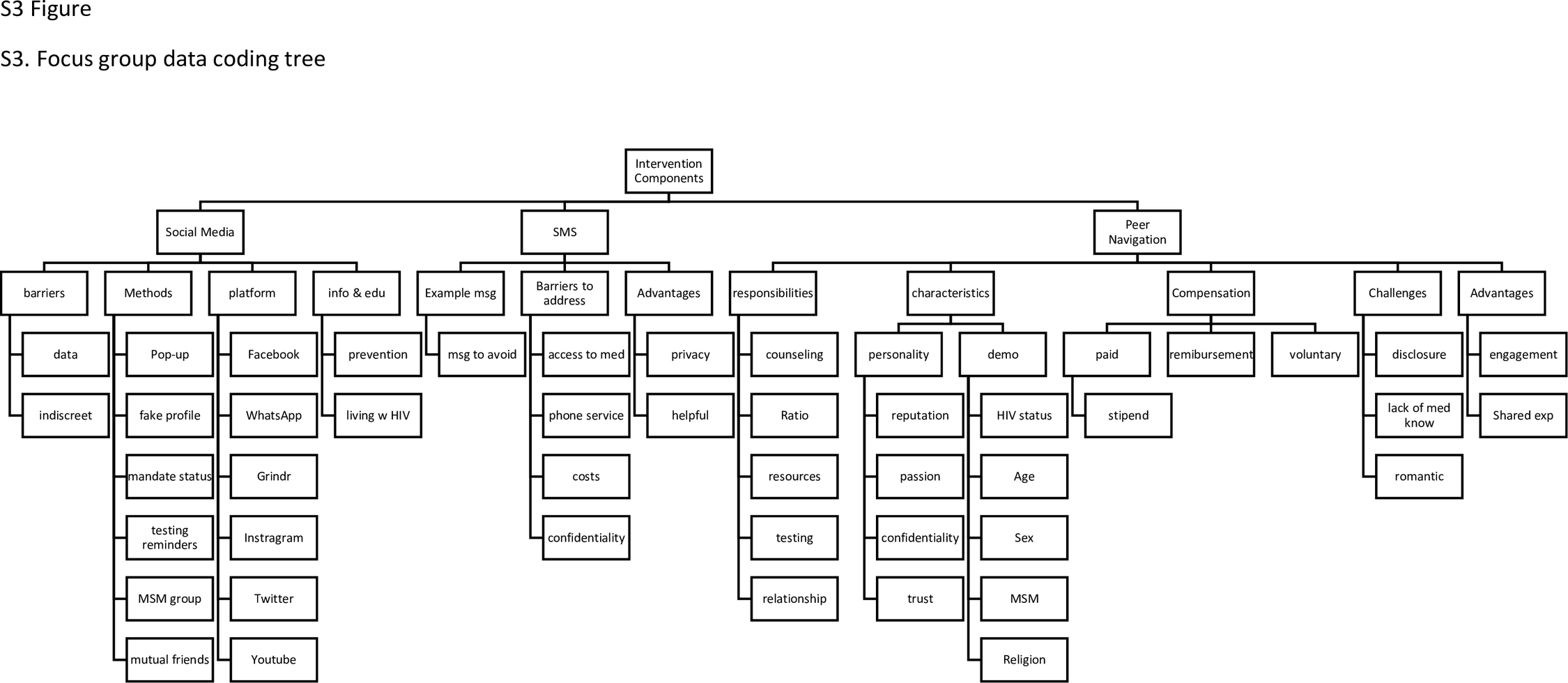

Supplement: S1 Fig — (TIF) [file pone.0258190.s003.tif]
